# Association between pregnancy intention and psychological distress among women exposed to different levels of restrictions during the COVID-19 pandemic in Australia

**DOI:** 10.1371/journal.pone.0273339

**Published:** 2022-08-25

**Authors:** Danielle A. J. M. Schoenaker, Christie Bennett, Jessica A. Grieger, Cheryce L. Harrison, Briony Hill, Joanne Enticott, Lisa J. Moran, Helena J. Teede, Sharleen L. O’Reilly, Siew Lim

**Affiliations:** 1 School of Primary Care, Population Sciences and Medical Education, Faculty of Medicine, University of Southampton, Southampton, United Kingdom; 2 NIHR Southampton Biomedical Research Centre, University of Southampton and University Hospital Southampton NHS Foundation Trust, Southampton, United Kingdom; 3 Department of Nutrition, Dietetics and Food, School of Clinical Sciences, Faculty of Medicine, Nursing and Health Sciences, Monash University, Clayton, VIC, Australia; 4 Robinson Research Institute, University of Adelaide, North Adelaide, SA, Australia; 5 Adelaide Medical School, University of Adelaide, Adelaide, SA, Australia; 6 Monash Centre for Health Research and Implementation, School of Public Health and Preventive Medicine, Monash University, Clayton, VIC, Australia; 7 Health and Social Care Unit, School of Public Health and Preventive Medicine, Monash University, Melbourne, Australia; 8 Department of Psychiatry, Southern Synergy, Monash University, Clayton, VIC, Australia; 9 UCD Institute of Food and Health, School of Agriculture and Food Science, University College Dublin, Belfield, Dublin, Ireland; Nazarbayev University School of Medicine, KAZAKHSTAN

## Abstract

**Background:**

The COVID-19 pandemic has had a negative impact on the mental health of people globally. Significant concerns about health and access to services among women of reproductive age considering pregnancy may cause psychological distress, and in turn increase health risks during and after pregnancy for mothers and offspring.

**Objectives:**

To examine the association between pregnancy intention and psychological distress during the COVID-19 pandemic in Australia, and explore if this association differed based on local viral transmission rates and corresponding levels of pandemic restrictions.

**Methods:**

A nationwide online survey was completed by 849 non-pregnant women aged 18–50 years between 15 October and 7 November 2020. Women were asked about their intention to become pregnant, and psychological distress was assessed using the Kessler Psychological Distress Scale (K10). Multivariable regression analysis examined associations between pregnancy intention and psychological distress. An interaction term was added to the model to examine differences in associations by level of viral transmission rates and lockdown restrictions which was determined based on postcode.

**Results:**

Pregnancy intention was not associated with experiencing (very) high psychological distress in the overall study population (odds ratio (OR) 1.42, 95% CI 0.94, 2.11). The interaction term (p = 0.09) suggested potential differences by level of restrictions and viral transmission rates. In stratified analysis among women living in a location with strict lockdown restrictions and high viral transmission rates leading up to and during the study, those planning to become pregnant were more likely to experience (very) high psychological distress (OR 3.39, 2.04, 5.65) compared with women not planning to become pregnant. Pregnancy intention was not associated with psychological distress among women exposed to lower levels of pandemic restrictions and viral transmission rates (OR 1.17, 0.74, 1.85).

**Conclusions:**

Our findings highlight the need to identify and support women planning pregnancy during a public health crisis to mitigate potential short- and long-term intergenerational negative health outcomes associated with psychological distress.

## Introduction

Maternal psychological distress, including non-specific prolonged stress, anxiety and/or depressive symptoms, can have profound effects on pregnancy, maternal health, and offspring development [1–3]. A growing body of evidence shows that moderate and severe psychological distress before and during pregnancy can increase the risk of adverse pregnancy outcomes including miscarriage, pre-eclampsia, gestational diabetes, low birth weight and preterm birth [1, 2]. Moreover, maternal exposure to distress can negatively influence the behavioural and physiological development of offspring across the life course [1, 3]. Psychological distress before pregnancy has been directly linked with adverse pregnancy and birth outcomes [2, 3], and may also be indirectly linked with these outcomes as psychological distress leading up to pregnancy often continues during pregnancy [1, 2]). Identifying risk factors for psychological distress among women of reproductive age who intend to become pregnant is therefore needed to inform strategies to prevent or manage psychological distress in this group.

While many risk factors for psychological distress have been studied including stressors related to early life trauma, interpersonal violence, family, life events and socioeconomic adversity [3–5], we are not aware of evidence on the association between pregnancy planning and non-specific psychological distress [6]. In a large cohort study of reproductive-aged Australian women surveyed in 2003 and 2009, higher levels of anxiety symptoms were associated with lower odds of aspiring to have a child among parous women but not among nulliparous women, and no associations were found for depressive symptoms [7]. These findings are based on a population-based cohort of mostly healthy women, and further studies are needed to examine associations between pregnancy intention and psychological factors in populations with potentially high levels of psychological distress [8].

In December 2019, the severe acute respiratory syndrome coronavirus (SARS-CoV-2) emerged and spread rapidly across the world. The coronavirus disease 2019 (COVID-19) pandemic has since led to large scale public health measures to limit the spread of the virus, including restrictions to people’s movements and activities. The negative impact of these measures on people’s mental health has been reported globally [9–13], and may be more pronounced in younger compared with older adults and in women compared with men, for example due to child-caring responsibilities [14–17]. Women who are planning to start or grow their family may have additional stressors associated with the COVID-19 pandemic, including anxiety about the potential effects of the virus on their fertility, pregnancy and baby, the changes in access to sexual and reproductive health and antenatal and birth services, and financial insecurity [18–21]. It is, however, not known if women planning a pregnancy during the COVID-19 pandemic experienced higher levels of psychological distress compared with women not planning pregnancy during the same time, and if this was influenced by local rates of viral transmission and the corresponding level of restrictions (i.e. type and amount of restrictions to people’s movements and activities). This evidence is needed to inform whether women planning pregnancy during the COVID-19 pandemic, and possible future public health crises, need additional support to optimise their psychological wellbeing before conception with the potential to reduce psychological distress related adverse health consequences for mothers and offspring.

The aim of the current study was to examine the association between pregnancy intention (exposure) and psychological distress (outcome) during the COVID-19 pandemic in Australia. We also aimed to explore whether this association differed based on local viral transmission rates and corresponding levels of pandemic restrictions.

## Materials and methods

### Study design and population

We conducted a cross-sectional study using data collected through an anonymous online survey using the Qualtrics platform between 15 October and 7 November 2020. Women of reproductive age (18 to 50 years) who resided in Australia were contacted via targeted emails by an external cross-panel market research provider using a well-established database and reimbursement in accordance with ISO 26362 and industry requirements. Participants were asked to complete an anonymous 10-minute online survey including a series of short answer and multiple-choice questions. To ensure broad representativeness of the study sample with the Australian population in terms of age and residential location (state/territory) according to the Australian Bureau of Statistics [22], these characteristics were examined after four days of recruitment and further sampling was targeted to underrepresented groups (based on distribution of age and residential location) until the target sample size of 1,000 respondents was reached. The study was approved by the Monash University Human Research Ethics Committee (MUHREC project: 25941). Participants provided online consent after reading the study purpose and prior to completing the online survey.

A total of 1,005 women completed the survey. For the current study, women were excluded if they were unsure or did not want to disclose their pregnancy intention (n = 74); were pregnant (n = 32); or had a baby in the last 12 months (n = 50). A total of 849 participants were included for analysis (S1 Fig).

### Level of viral transmission rates and corresponding lockdown restrictions

From mid-March 2020, Australia introduced restrictions to international travel from all countries as well as travel between states, large gatherings were banned, and non-essential shops, entertainment, food (excluding takeaway) and recreation venues closed to reduce transmission of the virus (S1 Table). In mid-May, community transmission was very low and most national restrictions were eased across the country except for the international travel restrictions. From this time, no further major restrictions were introduced across Australia in 2020, with the exception of metropolitan Melbourne [23]. The strict measures in place to contain the virus in metropolitan Melbourne continued until mid-October after which they were gradually eased by December 2020. As this nationwide cross-sectional study was conducted at the end of the lockdown in metropolitan Melbourne when restrictions started to ease (15 October and 7 November 2020), the localised differences in viral transmission rate and level of restrictions within Australia provide a unique natural experiment to compare pregnancy intentions and psychological distress between women living in metropolitan Melbourne compared with women living in other regions. S1 Table provides an overview of key public health policies implemented in Australia from the start of the pandemic until the time of the survey.

To examine the potential impact of the level of viral transmission rates and corresponding lockdown restrictions during the months leading up to the survey, location was dichotomised as exposure to high viral transmission rates and strict lockdown restrictions (i.e. women living in metropolitan Melbourne) vs exposure to low viral transmission rates and less strict lockdown restrictions (i.e. women living in any other region in Australia).

### Pregnancy intention

Women were asked *What is your intention around pregnancy*? with response options including “I am not planning to become pregnant”, “I am planning to become pregnant”, "I am currently pregnant", "I just had a baby in the last 12 months" and "I don’t know/I prefer not to answer". Women were categorised as “Planning to become pregnant” or “Not planning to become pregnant” based on the first two response options. Women who responded to the latter three options were excluded from the analyses.

### Psychological distress

The Kessler Psychological Distress Scale (K10) was used to assess psychological distress [24]. The K10 is a 10-item questionnaire to measure the level of distress based on questions about non-specific prolonged stress, anxiety and depressive symptoms that an individual has experienced in the most recent 4-week period. It has been widely used in national surveys, including in Australia [25, 26], and has established associations for higher scores with increasing prevalence of mental disorders [26, 27]. All 10 questions are answered using a five-point scale, with the total score ranging from 10 to 50. The K10 items demonstrated a Cronbach’s alpha of 0.93 in our study, indicating a high level of internal consistency. A score below 22 was categorised as low/moderate distress, and 22 or higher as high/very high distress [28].

### Assessment of population characteristics

The survey included multiple-choice questions to assess participants’ age group, cultural or ethnic group, highest level of education completed, employment status before the pandemic, changes in employment status during the pandemic, annual household income before tax, and marital status. Urban or rural/remote location was determined based on postcode. The survey also included questions on the number of children living in the household, household food affordability, alcohol consumption, and time spent doing moderate- and vigorous-intensity physical activities. Data on self-reported weight and height were used to calculate body mass index (BMI) and categorised as normal weight (<25 kg/m^2^, including n = 30 women (3.5%) with underweight), overweight (25–29.9 kg/m^2^) or obesity (≥30 kg/m^2^).

### Statistical analysis

Population characteristics were described for the overall study sample and according to pregnancy intention using percentages and compared using chi-square test. Characteristics of women with (n = 289) and without missing data on any variable (n = 560) were also described and compared using chi-square test.

Regression analyses were used to examine associations of pregnancy intention with psychological distress. Linear regression was used to analyse the association with psychological distress scores (log-transformed to improve normality of the distribution). Logistic regression was used for psychological distress dichotomised as low/moderate compared with high/very high distress. To explore differences in the association by level of lockdown restrictions, an interaction term was added to the model (pregnancy intention x level of restrictions) and stratified analyses were conducted if the interaction term p-value was less than 0.10. All models were adjusted for predetermined confounding factors known to be associated with both pregnancy planning and psychological distress, including age group, location (in non-stratified analyses), marital status, highest level of education completed, annual household income before tax, food affordability, employment (prior to the pandemic), number of children in the household, alcohol consumption and BMI [4–7, 13]. Variance inflation factors (VIF) were determined and <3 for all regression analyses, indicating no multicollinearity issues.

Compared with women without missing data, women with missing data did not differ in terms of location, remoteness, marital status, number of children in the household, psychological distress, physical health condition and alcohol consumption, however women with missing data were more likely to be younger, not plan to become pregnant, from an ethnic minority background, have lower education and income, be unemployed, and have a healthy weight (S2 Table). For the main analysis, we therefore performed multiple imputation using the MICE method to account for potential biases introduced by missing data [29]. The imputation models included variables for all characteristics described above (pregnancy intention, psychological distress, level of lockdown restrictions and population characteristics). Stata commands ‘mi impute’ and ‘mi estimate’ were used to generate, pool and analyse data from 20 imputed datasets [29]. Multiple imputation was applied for all regression analyses. As a sensitivity analysis, regression analyses without multiple imputation (complete case analysis) were performed.

All statistical analyses were conducted using Stata/SE 16 (StataCorp LLC). Statistical significance was defined at p <0.05.

## Results

### Study population

About half of the 849 non-pregnant women of reproductive age in this study were aged 35 to 50 years (51%), one in four lived in metropolitan Melbourne (24%) and the majority lived in an urban area (95%) (Table 1). Just over half of women identified as Australian, New Zealander or Pacific Islander (57%), while 25% identified as European or North American, and 12% as Asian. Half of women had a university or post-graduate degree (55%) and 48% had an annual household income before tax of AUD $100,000 or more (Table 1).

**Table 1:** Participant characteristics overall and according to pregnancy intention, n = 849^1^.

	All women n = 849	Not planning to become pregnant n = 693 (81.6%)	Planning to become pregnant n = 156 (18.4%)	
	n (%)	n (%)	n (%)	p-value^2^
***Socio-demographic characteristics***				
Age group				<0.0001
18–24 years	142 (16.7)	110 (15.9)	32 (20.5)	
25–34 years	274 (32.3)	185 (26.7)	89 (57.1)	
35–50 years	433 (51.0)	398 (57.4)	35 (22.4)	
Location				0.50
Victoria–Metropolitan Melbourne	203 (23.9)	161 (23.2)	42 (26.9)	
Regional Victoria, ACT, NT, WA, SA, TAS	219 (25.8)	184 (26.6)	35 (22.4)	
Queensland	170 (20.0)	142 (20.5)	28 (18.0)	
New South Wales	257 (30.3)	206 (29.7)	51 (32.7)	
Remoteness				0.04
Rural or remote	45 (5.3)	42 (6.1)	3 (1.9)	
Urban	804 (94.7)	651 (93.9)	153 (98.1)	
Cultural or ethnic group				0.68
Oceanian	481 (56.7)	395 (57.0)	86 (55.1)	
European or North American	209 (24.6)	170 (24.5)	39 (25.0)	
Asian	99 (11.7)	77 (11.1)	22 (14.1)	
Other^3^	60 (7.1)	51 (7.4)	9 (5.8)	
Marital status				0.06
Single	373 (43.9)	315 (45.5)	58 (37.2)	
Married or de facto	476 (56.1)	378 (54.6)	98 (62.8)	
Highest level of education completed				0.001
Primary, secondary or high school	177 (20.9)	154 (22.3)	23 (14.7)	
Diploma or certificate (TAFE)	205 (24.2)	179 (25.9)	26 (16.7)	
University or post-graduate degree	464 (54.9)	357 (51.7)	107 (68.6)	
Annual household income before tax				0.02
AUD $0 to 99,999	382 (51.6)	322 (53.8)	60 (42.6)	
AUD ≥ $100,000	358 (48.4)	277 (46.2)	81 (57.5)	
Not able to afford balanced meals for household				0.23
Never true	641 (75.5)	529 (76.3)	112 (71.8)	
Sometimes or often true	208 (24.5)	164 (23.7)	44 (28.2)	
Employment prior to the pandemic				<0.0001
Unemployed	202 (23.8)	183 (26.4)	19 (12.2)	
Full-time employment	419 (49.4)	310 (44.7)	109 (69.9)	
Part-time or casual employment	228 (26.9)	200 (28.9)	28 (18.0)	
Changes in employment since the pandemic				0.25
No change or change between part-time and full-time	781 (92.0)	641 (92.5)	140 (89.7)	
Change from employed to unemployed	68 (8.0)	52 (7.5)	16 (10.3)	
Number of children in the household				0.002
None	511 (60.3)	400 (57.8)	111 (71.2)	
One or more	337 (39.7)	292 (42.2)	45 (28.9)	
***Health and health behaviours***				
Psychological distress				0.002
Low or moderate	487 (58.5)	415 (61.0)	72 (47.1)	
High or very high	346 (41.5)	265 (39.0)	81 (52.9)	
Common physical health condition, such as diabetes, hypertension, polycystic ovary syndrome				0.73
Yes	227 (26.7)	187 (27.0)	40 (25.6)	
No	622 (73.3)	506 (73.0)	116 (74.4)	
Body mass index^4^				0.05
Normal weight (<25 kg/m^2^)	362 (58.2)	278 (55.8)	84 (67.7)	
Overweight (25–29.9 kg/m^2^)	136 (21.9)	113 (22.7)	23 (18.6)	
Obesity (≥30 kg/m^2^)	124 (19.9)	107 (21.5)	17 (13.7)	
Any alcohol consumption				0.01
No	191 (22.5)	168 (24.2)	23 (14.7)	
Yes	658 (77.5)	525 (75.8)	133 (85.3)	
≥ 30 minutes of moderate- or vigorous-intensity physical activity per day				0.51
Yes	269 (31.7)	223 (32.2)	46 (29.5)	
No	580 (68.3)	470 (67.8)	110 (70.5)	

ACT, Australian Capital Territory; NT, Northern Territory; SA, South Australia, TAS, Tasmania; WA, Western Australia.

^1^ Number of participants differs due to missing data.

^2^ p value from chi-square test.

^3^ Other cultural or ethnic groups include African, Middle Eastern, South American, Central American and Caribbean Islander.

^4^ Women with underweight (BMI <18.5 kg/m^2^) were included in the normal weight category due to low numbers (n = 30).

Overall, 18.4% of women (n = 156) reported they planned to become pregnant. This proportion did not differ across states, with 20.7% of women in Metropolitan Melbourne, 16.5% in Queensland, 19.8% in New South Wales, and 16.0% in regional Victoria and other states reporting planning to become pregnant (p = 0.50). Compared with women not planning pregnancy, women planning to become pregnant were more likely to be aged 25–34 and less likely to be 35–50 years, more likely to live in an urban area, to be married or in a de facto relationship, to have a university or post-graduate degree, to have a higher income, to be in full-time employment, to not have children living in the household, and to consume alcohol (Table 1). Among all women, more than four in 10 reported high/very psychological distress (42%). Women planning to become pregnant were more likely to experience high/very high psychological distress compared with women not planning pregnancy (53% vs 39%, p = 0.002) (Table 1).

### Associations between pregnancy intention and psychological distress

In unadjusted analysis, women planning pregnancy had higher log-transformed psychological distress scores (β 0.11, 95% CI 0.04, 0.18) and were more likely to have high/very high psychological distress (odds ratio (OR) 1.74, 95% CI 1.23, 2.47) compared with women not planning to become pregnant (Table 2). These overall associations were attenuated and no longer statistically significant after adjustment for confounders (β 0.05, 95% CI -0.02, 0.12; OR 1.42, 95% CI 0.94, 2.11).

**Table 2:** Associations between pregnancy intention and psychological distress by level of COVID-19 transmission rates and lockdown restrictions, N = 849.

		Outcome: log-transformed psychological distress score		Outcome: high/very high vs low/moderate psychological distress	
	n (%)	Unadjusted Coefficient (95% CI)	Adjusted^1^ Coefficient (95% CI)	Unadjusted Odds ratio (95% CI)	Adjusted^1^ Odds ratio (95% CI)
*Overall study population*, *n = 849*					
Not planning to become pregnant	693 (81.6)	Reference	Reference	Reference	Reference
Planning to become pregnant	156 (18.4)	**0.11 (0.04, 0.18)**	0.05 (-0.02, 0.12)	**1.74 (1.23, 2.47)**	1.42 (0.94, 2.11)
*Women exposed to high viral transmission rates and strict lockdown restrictions*, *n = 203*					
Not planning to become pregnant	161 (79.3)	Reference	Reference	Reference	Reference
Planning to become pregnant	42 (20.7)	**0.22 (0.08, 0.35)**	**0.20 (0.05, 0.34)**	**3.32 (2.16, 5.11)**	**3.39 (2.04, 5.65)**
*Women exposed to low viral transmission rates and less strict lockdown restrictions*, *n = 646*					
Not planning to become pregnant	532 (82.4)	Reference	Reference	Reference	Reference
Planning to become pregnant	114 (17.6)	0.06 (-0.02, 0.15)	0.02 (-0.08, 0.10)	1.39 (0.93, 2.09)	1.17 (0.74, 1.85)

Results are based on imputed data where missing data were imputed using the MICE method [29].

^1^ Adjusted for age group, marital status, location (overall study population only), highest level of education completed, annual household income before tax, food affordability, employment prior to the pandemic, number of children in the household, alcohol consumption, body mass index.

P-values for interaction terms were 0.07 and 0.09 for associations of pregnancy intention with psychological distress scores and high/very high psychological distress, respectively, suggesting potential differences by level of lockdown restrictions. Stratified analyses showed women planning pregnancy (compared with not planning pregnancy) reported higher psychological distress scores if they were exposed to high viral transmission rates and strict lockdown restrictions (β 0.20, 95% CI 0.05, 0.34), but not if they lived under less strict measures (β 0.02, 95% CI -0.08, 0.10), after adjustment for confounders (Table 2). Similarly, women planning pregnancy were more likely to experience high/very high psychological distress if they were exposed to high viral transmission rates and strict lockdown restrictions (OR 3.39, 95% CI 2.04, 5.65), but not if they lived under less strict restrictions (OR 1.17, 95% CI 0.74, 1.84) (Table 2).

Conclusions based on results from complete case analysis (non-imputed data) were comparable (S3 Table).

## Discussion

In this large survey conducted during the COVID-19 pandemic among non-pregnant women of reproductive age in Australia, we found no overall association between pregnancy intention and psychological distress. However, our findings suggested the relationship between pregnancy intention and psychological distress differs based on local viral transmission rates and level of restrictions. Among women who lived in Metropolitan Melbourne following an extended period of high viral transmission rates and strict lockdown measures, those who planned to become pregnant were more likely to experience high/very high psychological distress compared with women not planning pregnancy. Among women living in other parts of Australia with lower rates of viral transmission and less strict restrictions, pregnancy intention was not associated with psychological distress.

Our findings build on the very limited evidence from previous studies on the association between pregnancy intention and psychological distress, and confirm the hypothesis that pregnancy intention may be related to poorer mental health only in populations at higher risk or with higher levels of psychological distress [8]. Similar to findings from an Australian population-based cohort conducted before the COVID-19 pandemic in 2003 and 2009 [7, 8], we found no association between pregnancy intention and psychological distress in the overall study population and among women exposed to low levels of pandemic restrictions. However, findings from the current study demonstrate that women exposed to strict lockdown measures and high viral transmission rates who planned to become pregnant experienced higher levels of psychological distress. This is in line with findings from a study conducted between 1996 and 1999 among 460 high-risk African American adolescents living in deprived areas of Birmingham, Alabama, which showed those who expressed a desire to become pregnant were more likely to report depressive symptoms (OR 1.7; 95% CI 1.2, 2.4) compared with their peers without pregnancy desire [30]. The reasons for the increased psychological distress among women who plan to become pregnant likely differ among African American women living in deprived communities and women living in metropolitan Melbourne during the strict lockdown. The factors causing psychological distress among women planning pregnancy in different circumstances should be explored to inform support services tailored to women’s concerns and needs.

Women’s decisions and preferences during the pandemic about timing of pregnancy have been studied in a number of surveys published in 2020 and 2021, and vary largely between populations and countries [18–21, 31–33]. In most studies, at least one-third of women reported deliberately postponing pregnancy plans, while smaller proportions had brought their pregnancy plans forward [18, 20, 21, 31–33]. This pattern of results was stronger among women with immediate pregnancy intentions compared with women with a longer-term desire to have a family [21] and among women from more deprived backgrounds [31]. Moreover, a survey conducted in five European countries in March and April 2020 suggested that the proportion of women postponing or abandoning their pregnancy plans may be higher in countries and regions with a higher prevalence of COVID-19 cases and with more frequent or longer lockdowns [32]. The most common reasons for postponing pregnancy plans that have been reported include concerns and anxiety about changes in pregnancy care, about the impact of COVID-19 on pregnant women and infants, and about changing life circumstances such as delayed marriage, career interruptions and financial insecurity [18–21, 34]. Moreover, women have reported difficulty accessing services to remove contraceptive devices or to provide fertility treatment [20]. Similar to previous public health epidemics (such as the Spanish flu and Zika virus) [35, 36] and economic crises [37, 38] which have increased uncertainties and anxiety related to pregnancy, the COVID-19 pandemic may be associated with delayed reproductive commitments and decreased fertility rates [39]. Although these studies have not directly compared women with and without pregnancy intentions in relation to psychological distress, the reasons reported for changes in pregnancy intentions all relate to increased distress and may explain our finding that women who plan to become pregnant while being exposed to high viral transmission rates and strict lockdown measures have higher levels of psychological distress.

Together with our findings, this evidence highlights the urgent need to identify and better support the well-being of women planning pregnancy during a crisis such as the COVID-19 pandemic. Provision of preconception care is increasingly appreciated as a means to improve the short- and long-term physical and mental health and health behaviours of women planning pregnancy [40–42], and would be a vital healthcare service during a public health crisis. Safe access to primary care, sexual and reproductive health and antenatal and birth services, and timely adaptation of (remote) service delivery depending on pandemic restrictions, should be a government priority [43]. Moreover, to address women’s concerns about a potential future pregnancy, clear and up-to-date public health messaging about the virus in relation to its impact on health of pregnant women and infants must be in place [44, 45]. At the time our study was conducted, the large-scale roll-out of the COVID-19 vaccine had not yet started, and going forward clear messaging that addresses any concerns or misconceptions about the effects of the vaccine on women’s fertility would be crucial to prevent psychological distress [46]. Monitoring of mental health trends among women planning pregnancy during and beyond the pandemic is also critical, to enable evaluation of the impact of preventive efforts [47, 48].

Limitations of our study should be considered when interpreting the results. Firstly, women’s pregnancy intentions were assessed by asking women “What is your intention around pregnancy?” with answer options relating to planning to become pregnant or not. This is not a validated measure, and the question and response options may have been interpreted differently between women in our study population as it does not include a timeframe to distinguish between an immediate or longer-term desire to become pregnant. Our finding that 37% of women who indicated they plan to become pregnant were single at the time of the survey suggests that for at least a proportion of women the intentions may relate to the longer-term desire to become pregnant. However, restricting our sample to 476 women who were married or de facto (excluding 373 women who were single) did not change the conclusions. This cross-sectional survey was also not originally designed to examine the influence of (changes in) level of pandemic restrictions on pregnancy intentions in relation with psychological distress, no data were available on COVID-19 infection among participants, and subgroup analyses were based on smaller groups of women. Due to the cross-sectional design of the study, we cannot exclude the possibility of reverse causation, and our findings need to be confirmed in prospective longitudinal studies. Moreover, we determined level of pandemic restrictions based on location. Although there was a clear distinction in level of viral transmission rates and lockdown restrictions in metropolitan Melbourne compared with all other regions in Australia during the months leading up to and during the survey, other factors that are related to living in metropolitan Melbourne, to pregnancy intention and to psychological distress may explain our findings. Our study population was broadly representative of the Australian population of women of reproductive age in terms of age distribution and residential location (state/territory), however, findings may not be fully generalisable due to limitations associated with participant recruitment through an external cross-panel market research provider, the high non-response rate, and data collection through online-only surveys. The possibility of residual confounding was minimised by adjustments for a range of factors including socio-economic factors, number of children in the household and health behaviours, however, lack of adjustment for potential key confounders that were not collected such as pre-existing mental health conditions is a limitation of our study and may have influenced our findings.

## Conclusion

In this study we found that, overall, women in Australia planning pregnancy during the COVID-19 pandemic did not have higher psychological distress compared with women not planning pregnancy. However, women planning pregnancy while living in a location with strict COVID-19 lockdown restrictions and high viral transmission rates reported substantially elevated psychological distress compared to women not planning pregnancy. Given the known effects of distress on pregnancy, infant and child outcomes, there is an urgent need to allocate resources to identify and support women planning pregnancy during public health crises to mitigate the short- and long-term intergenerational negative outcomes. While national population-wide mental health strategies are needed during the COVID-19 pandemic and its recovery [49], women planning pregnancy will need support strategies that are tailored to their specific concerns around their future pregnancy and family. Health services and public health messages that address these concerns may contribute to reducing psychological distress and adverse outcomes among women who become pregnant and their offspring.

## Supporting information

S1 Fig Flow chart describing in- and exclusion of study participants. (DOCX)

S1 Table Australian Federal, State and Territory policies and regulations in response to COVID‐19. (DOCX)

S2 Table Comparison of characteristics of women with and without missing data. (DOCX)

S3 Table Associations between pregnancy intention and psychological distress by level of COVID-19 transmission rates and lockdown restrictions based on complete case analysis (non-imputed data), N = 560. (DOCX)

S1 File Copy of the survey questionnaire. (PDF)
