# Supplementary figures and images for: Association between pregnancy intention and psychological distress among women exposed to different levels of restrictions during the COVID-19 pandemic in Australia
Source: PLoS One. 2022 Aug 25;17(8):e0273339. doi: 10.1371/journal.pone.0273339 (PMC9409515; doi:10.1371/journal.pone.0273339)

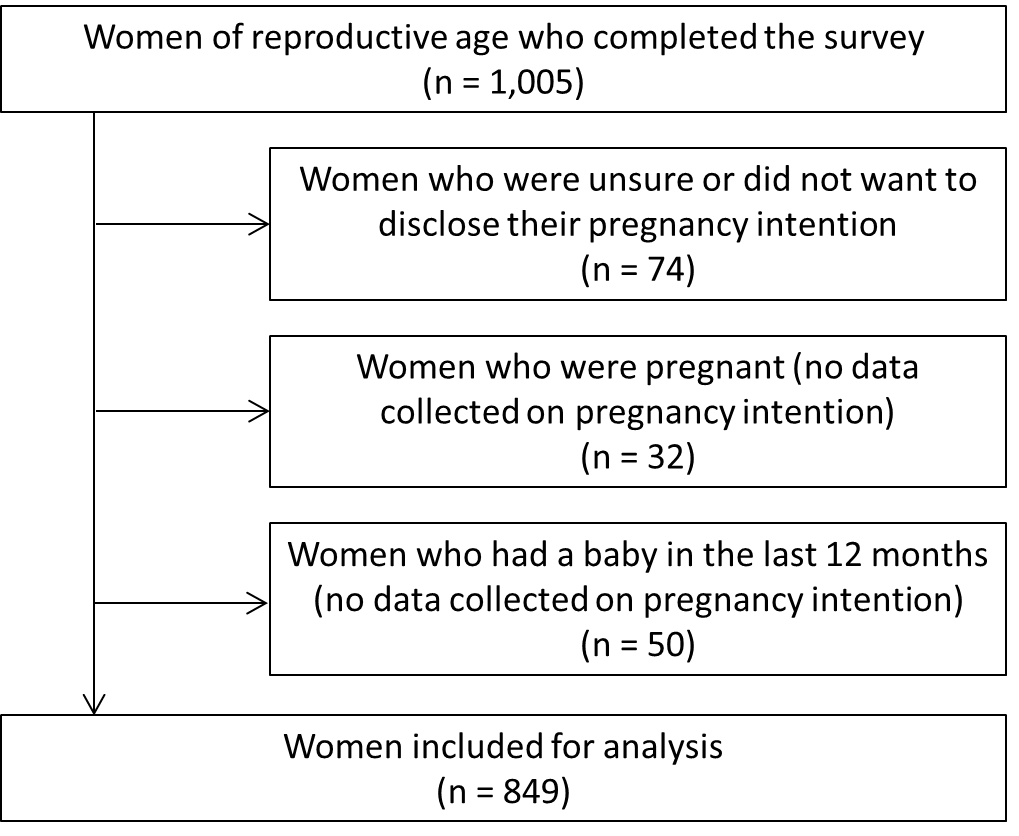


**S1 Figure.** Flow chart describing in- and exclusion of study participants

Supplement: S1 Fig — (DOCX) [file pone.0273339.s001.docx]
