# Supplementary material for: Association between pregnancy intention and psychological distress among women exposed to different levels of restrictions during the COVID-19 pandemic in Australia
Source: PLoS One. 2022 Aug 25;17(8):e0273339. doi: 10.1371/journal.pone.0273339 (PMC9409515; doi:10.1371/journal.pone.0273339)
Supplement: S1 Table — (DOCX) [file pone.0273339.s002.docx]

**S1 Table.** Australian Federal, State and Territory policies and regulations in response to COVID‐19^1^

| **Date** | **Policy measure** | **Further details** |
| --- | --- | --- |
| **Travel restrictions** | | |
| 01/02/2020 | - First ban on foreign nationals entering Australia from nominated countries - Australian residents and citizens not allowed to travel to nominated country - Returning travellers from nominated countries required to self-isolate at home for 14 days | Specific dates when countries banned:   - 1st February: China - 1st March: Iran - 5th March: Korea - 11th March: Italy - 18th March: All Countries |
| 15/03/2020 | - Return travellers from ALL countries required to self‐isolate at home for 14 days - Ban on cruise ships from foreign ports |  |
| 18/03/2020 | - All outbound and in‐bound travel banned - Australian citizens and residents told to return home or risk being unable to | Exception: “essential” travel including humanitarian and medical reasons |
| 19/03/2020 | - First interstate border‐restrictions: Tasmania closes borders to all “non‐essential travellers” unless they undertake mandatory 14-day quarantine | Subsequent border restrictions:   - 24^th^ March – Northern Territory, Western Australia and South Australia - 11^th^ April – Queensland - 8^th^ July ‐ Victorian‐NSW border closed   Subsequent easing/tightening of border restrictions between states has occurred on a discretionary basis depending on case numbers |
| 27/03/2020 | - Australian Mandatory Hotel Quarantine Program Introduced |  |
| 30/06/2020 | - All flights to Victoria diverted to other Australian cities temporarily |  |
| **Physical, social and event restrictions** | | |
| 16/03/2020 | - Ban on non‐essential, organised gatherings of >500 people | Exceptions: Schools, universities, workplaces, public transport, domestic travel and public transient places |
| 18/03/2020 | - Ban on non‐essential indoor gatherings of > 100 people - Physical distancing of 1.5 metres introduced in all settings | Exceptions: Public transport, medical/ health care facilities, pharmacies, emergency services, correctional facilities,  youth justice and some others |
| 20/03/2020 | - For gatherings <100 people, floor space of 1 person per 4 square metres required with at least 1.5 metres distance between people |  |
| 30/03/2020 | - Stay at home requirements introduced | Exceptions to “stay at home requirement”:  1. medical or healthcare needs  2. daily exercise  3. work or study if unable to complete at home  4. shopping for essential items |
|  | - Indoor and outdoor gatherings restricted to two people only | Each State and Territory applied this rule separately (as examples, Victoria banned all non‐essential social visits, whereas Queensland later allowed two known  visitors to each household) |
| 8/5/2020 | - National three stage framework to lift restrictions announced | Several regions began lifting restrictions earlier:   - 27 April, Western Australia, allowed 10 people at some indoor and outdoor non‐work gatherings - 1^st^ May, NSW allowed two adults to visit another household |
| 15/5/2020 | - QLD and NT further ease restrictions in accordance with national framework |  |
| 31/5/2020 | - Victoria steps down from stage 3 restrictions | 1) Increase to 20 people at household/outdoor gatherings; 2) Increase to 20 people at weddings or religious ceremonies, up to 50 people at a funeral; 3) Entertainment, cultural venues, swimming pools to re-open; 4) Auctions & open for inspections subject to 20  person limit; 5) Next set of easing restrictions from 22 June |
| 22/6/2020 | - Victoria cancels plans to ease restrictions, tightening social measures in light of rising case numbers | Households only allowed to have five visitors at home Outside gatherings of families and friends will be limited to 10 people |
| 08/07/2020 | - Stage 3 restrictions applied to Melbourne and Mitchel shire following beginning of second wave | Metropolitan Melbourne will no longer be allowed to leave their homes except for grocery shopping, caregiving, exercise or work |
| 10/07/2020 | - Facemasks recommended in Melbourne when not at home - Made compulsory in Melbourne on 22^nd^ July - Recommended in Victoria on 30^th^ July - Made compulsory in all Victoria on 2^nd^ August |  |
| 02/08/2020 | - Tightening of restrictions to contain 2^nd^ Wave   1) Victorian state of disaster declared  2) Stage 4 restrictions for Melbourne  3) Stage 3 restrictions for regional Victoria  4) All schools return to remote learning | 2) stage 4 details: curfew announced from 8 pm to 5am, shopping for essential items must only be done within a 5 km radius with only one person per household allowed to shop per day, exercise within  five kilometres of the home for one hour maximum, once daily; outdoor gatherings limited to two people  4) kindergarten/ childcare only open for children of vulnerable/essential workers in metropolitan Melbourne, will remain open to all in regional areas |
| 06/09/2020 | - Victorian government announced roadmap for easing restrictions tied to case numbers |  |
| 18/10/2020 | - Regional Victoria was moved to "Step Three", which included the reopening of most businesses to the public, increased seating for hospitality, the allowance of visitors for all residents and the resumption of some indoor sports. - Metropolitan Victoria was increased to 25 km and the two-hour time limit was removed, however the border between metropolitan and regional Victoria was strengthened with extra checkpoints added |  |
| 26/10/2020 | - Victoria: Second wave ended with zero new cases being recorded | Retail, restaurants, cafes and bars to open up in Melbourne. Gatherings of up to ten people outdoors are now allowed from any number of households. |
| 8/11/2020 | - Further restrictions in Victoria eased, now in line with the rest of the Australian states | The 25 km travel limit in Greater Melbourne, and the border between Melbourne and regional Victoria was lifted; gyms and fitness studios were allowed to reopen; and the patron limit at restaurants, cafes and bars was raised. |
| 16/11/2020 | - Significant restrictions reintroduced in South Australia after an outbreak of coronavirus in the northern suburbs of Adelaide | 6-day lockdown |
| 21/11/2020 | - Restrictions ended for South Australia |  |
| **Business restrictions** | | |
| 23/03/2020 | - Initial restrictions on businesses set | Some non‐essential venues closed, including hotels (excluding accommodation), clubs, gyms, indoor  sporting venues, cinemas, entertainment venues, casinos, night clubs, religious gatherings, places of worship or funerals, except very small groups (observing previous social distancing rules).  Restaurants and cafes open for take‐away or delivery  only. |
| 25/03/2020 | - Further restrictions on businesses set | Most non‐essential venues closed, including but not limited to, beauty therapy, tanning, waxing, nail salons, tattoo parlours, spas, massage parlours, play centres, amusement parks, health clubs, museums, libraries, pools, youth centres and community facilities.  Hairdressers, boot camp and personal trainings (maximum 10 people) remain open.  Weddings (maximum 5 people), funerals (maximum 10 people) with social distancing |
| 31/5/2020 | - Victoria eases stage three business restrictions |  |
| 14/06/2020 | - Victorian announces that business restrictions will ease further   from the 21^st^ of June | Plan for libraries & community halls to open up to 50 people; recommencement of sports etc |
| 21/06/2020 | - Victoria eases restrictions for gyms, cinemas etc as planned, with a   few exceptions | 20-person limit to remain for all businesses, despite initial plan to allow 50 people |
| 06/08/2020 | - Victorian business closures for at least 6 weeks across retail, administration and some manufacturing | Some stores to remain operational via delivery and “click and collect” |
| 02/09/2020 | - Australian economy went into recession | Country's gross domestic product (GDP) fell 7% in the June quarter |
| 13/09/2020 | - All jurisdictions have requirements for either a COVIDSafe Plan or a COVID Safety | Mandatory for every Victorian business to have a COVID Safe Plan. High Risk industries must create a ‘HIGH RISK COVIDSafe plan.  Other state governments to provide sector-based guidance, including checklists, for COVID Safety Plans for business |
| 01/12/2020 | - Australia had pulled out of recession | 3.3% growth in GDP in the September quarter |
| **Punitive measures – fines, arrest, penalties** | | |
| 16/03/2020 | - States begin introducing fines for:   - failing to self‐isolate   - breaching event restrictions | $11,000 ‐ $50,000 depending on state, possible prison sentences |
| 27/3/2020 | - Queensland announced fines for deliberately coughing or spitting on someone | $13,345 per person |
| 29/03/2020 | - Victoria announces fines for breaches including gatherings, failure to quarantine, business regulations etc. | $1652 for non‐essential travel  $9,913 for businesses |
| 18/07/2020 | - Victoria: $200 fine for not wearing mask |  |
| 17/07/2020 | - New fines for venues breaching COVID safe restrictions/regulations in some states | Up to $55,000 per venue, plus $27,500 for every additional day the breach is continued |
| 04/08/2020 | - Victoria announces spot fines for breaching lockdown rules | $4,957 per person |
| **Financial support for business** | | |
| 12/03/2020 | - Increased instant asset write‐off (IAWO) threshold from $30, 000 to $150, 000 | Government to provide up to $100, 000 to eligible small and medium‐sized businesses and not‐for-profits; with a minimum payment of $20,000. |
| 22/03/2020 | - Increased cash flow for employers |  |
| 30/03/2020 | - Wage subsidy scheme introduced for eligible employers | JobKeeper payment introduced ‐ $1500 payment per fortnight (before tax) for each eligible employee/ self-employed individual for up to six months |
| **Financial support for individuals and households** | | |
| 12/03/2020 | - First $750 payment to eligible recipients announced, to be delivered by 13^th^ April | Available to social security, veteran and other income support recipients and eligible concession card holders |
| 22/03/2020 | - Second $750 payment to eligible recipients announced, to be delivered on 10^th^ July |  |
|  | - Coronavirus supplement announced | Eligible income support recipients receive $550 per fortnight for six months from 27/4/20 in addition to JobSeeker allowance |
| 24/03/2020 | - JobSeeker and Youth Allowance income support payment eligibility expanded | Eligible recipients include newly unemployed sole traders, self‐employed, casual workers, contract workers, eligible individuals caring for someone affected by coronavirus |
| 29/03/2020 | - Temporary moratorium on evictions from rental properties for people facing financial distress |  |
| 22/07/2020 | - Victoria announces payment to assist those awaiting test results   - $300 whilst awaiting test results, if unable to work   - $1500 whilst self‐isolating for two weeks, if unable to work | $300 payment increased to $450 on the 13^th^ of August |
| **School and education centres** | | |
| 22/03/2020 | - Federal Government encourages schools to remain open until term 1 break | Each State and Territory applied this rule separately (some commenced Term 1 break early on 24^th^ March) |
| 06/04/2020 | - Families receive free childcare |  |
| 16/04/2020 | - Federal Government advises schools to open for Term 2, but individual states apply discretion | E.g. South Australia opened schools for Term 2, New South Wales and Victoria announced a staggered plan to return students with first students returning on 11^th^ and 26^th^ of May respectively |
| 08/7/2020 | - Victoria: All year levels except year 11 and 12 delayed return to school from holidays for 1 week |  |
| 02/8/2020 | - Victoria: All students return to remote learning |  |
| **Healthcare policies** | | |
| 13/03/2020 | - New temporary Medicare Benefits Schedule (MBS) Telehealth numbers to support free telehealth medical consultations | For consultations for individuals at risk of COVID‐19 infection or those who have self‐isolated as a result of COVID‐19 (13^th^ March), expanded to all Medicare-eligible^2^ Australians (30^th^ March) |
| 18/03/2020 | - Aged care visitor restrictions introduced for:   - More than 2 people   - Anyone with contact with COVID‐19   - Anyone with symptoms | Exceptions: Discretionary groups allowed for end of life situations |
| 19/03/2020 | - Limited prescription medication purchasing to avoid stockpiling |  |
| 25/03/2020 | - Temporary suspension of all semi‐urgent elective surgery in the public hospital and private hospital system (1^st^ April). | Policy eased 27^th^ April |
| 29/03/2020 | - Funding announced for domestic violence, mental health support and for other vulnerable Australians |  |
| 09/08/2020 | - Victoria: $60 million for mental health support announced |  |
| **Testing** | | |
| 10/03/2020 | - South Australia and Victoria open first “drive through” testing clinics - $205 Million project announced to establish 100 “pop‐up” testing clinics across Australia | Other states quickly follow:   - NSW – 12th March - QLD – 14th March - ACT – 19th March   COVID‐19 tests free for everyone |
| 26/02/2020 | - Expanded national minimum testing criteria | Testing criteria expanded to include people with fever or acute respiratory infection in at‐risk categories including, but not limited to, all health workers, all aged/residential workers, hospitalised patients with  symptoms of unknown cause, geographically localised areas where there is elevated risk or high‐risk settings. |
| 24/04/2020 | - Expanded national minimum testing criteria to include mild symptoms | Testing to include all people with mild symptoms of COVID‐19 |
| 14/06/2020 | - Victoria announces targeted testing strategy to end of August | Targeting local government areas with low testing rates, communities with high case numbers, high‐risk workforces and vulnerable groups |
| 25/06/2020 | - Victoria announces testing blitz of 10 “priority” suburbs in Melbourne | Asymptomatic testing in chosen areas |
| 28/06/2020 | 1. National mandatory testing on day 3 and 11 for all returned travellers in hotel quarantine  2. Introduction of saliva sample test to increase testing uptake in prescribed circumstances | 1. Those who refuse to comply are forced to remain in hotel quarantine for an additional 10 days |
| **Self‐quarantine and self‐isolation** | | |
| 15/03/2020 | - For all returned travellers from all countries, 14 days |  |
| 26/03/2020 | - For confirmed cases and those who suspect they have COVID‐19 or   have symptoms, 14 days | To be nationally consistent at the State and Territory level |
| **Contract tracing** | | |
| 26/04/2020 | - Voluntary tracking and tracing app released | COVIDSafe App aims to assist the Government in contact tracing, by recording close contact between two users of the app |
| 01/06/2020 | - Some states introduce mandatory “check‐in” details for customers in restaurants, cafes etc. |  |
| 18/09/2020 | - Nationally consistent approach to contact tracing announced |  |

This table is modified with permission from Enticott J et al. *Frontiers in Public Health*. 2021. Doi: 10.3389/fpubh.2021.630189

**^1^** From February to April, Federal policies are emphasised. From April/June onwards, Victorian and New South Wales policies are emphasised as the second wave was localised to these states.

^2^ Medicare is Australia’s health care that enables free or lower cost community health care and free hospital-based health services. It covers Australian and New Zealand citizens, permanent residents and some others. <https://www.healthdirect.gov.au/what‐is‐medicare>
