# Supplementary material for: Association between pregnancy intention and psychological distress among women exposed to different levels of restrictions during the COVID-19 pandemic in Australia
Source: PLoS One. 2022 Aug 25;17(8):e0273339. doi: 10.1371/journal.pone.0273339 (PMC9409515; doi:10.1371/journal.pone.0273339)
Supplement: S2 Table — (DOCX) [file pone.0273339.s003.docx]

**S2 Table.** Comparison of characteristics of women with and without missing data

| Characteristic | Missing data  (n = 289^1^)  % | No missing data  (n = 560)  % | p-value^2^ |
| --- | --- | --- | --- |
| Age group |  |  | 0.03 |
| 18-24 years | 19.4 | 15.4 |  |
| 25-34 years | 26.6 | 35.2 |  |
| 35-50 years | 54.0 | 49.5 |  |
| Location |  |  | 0.13 |
| Metropolitan Melbourne | 27.0 | 22.3 |  |
| Other | 73.0 | 77.7 |  |
| Pregnancy intention |  |  | 0.02 |
| Not planning to become pregnant | 85.8 | 79.5 |  |
| Planning to become pregnant | 14.2 | 20.5 |  |
| Remoteness |  |  | 0.16 |
| Rural or remote | 3.8 | 6.1 |  |
| Urban | 96.2 | 93.9 |  |
| Cultural or ethnic group |  |  | 0.004 |
| Oceanian | 55.4 | 57.3 |  |
| European or North American | 24.9 | 24.5 |  |
| Asian | 8.7 | 13.2 |  |
| Other^3^ | 11.1 | 5.0 |  |
| Marital status |  |  | 0.08 |
| Married or de facto | 51.9 | 58.2 |  |
| Single | 48.1 | 41.8 |  |
| Highest level of education completed |  |  | <0.0001 |
| Primary, secondary or high school | 27.6 | 17.5 |  |
| Diploma or certificate (TAFE) | 26.2 | 23.2 |  |
| University or post-graduate degree | 46.2 | 59.3 |  |
| Annual household income before tax |  |  | 0.04 |
| AUD $0 to 99,999 | 58.3 | 49.5 |  |
| AUD ≥ $100,000 | 41.7 | 50.5 |  |
| Not able to afford balanced meals for household |  |  | <0.0001 |
| Never true | 66.4 | 80.2 |  |
| Sometimes or often true | 33.6 | 19.8 |  |
| Employment prior to the pandemic |  |  | <0.0001 |
| Unemployed | 32.9 | 19.1 |  |
| Full-time employment | 40.5 | 53.9 |  |
| Part-time or casual employment | 26.6 | 27.0 |  |
| Changes in employment since the pandemic |  |  | 0.76 |
| No change or change between part-time and full-time | 92.4 | 91.8 |  |
| Change from employed to unemployed | 7.6 | 8.2 |  |
| Number of children in the household |  |  | 0.71 |
| None | 59.4 | 60.7 |  |
| One or more | 40.6 | 39.3 |  |
| ***Health and health behaviours*** |  |  |  |
| Psychological distress |  |  | 0.15 |
| Low or moderate | 55.0 | 60.2 |  |
| High or very high | 45.1 | 39.8 |  |
| Physical health condition |  |  | 0.06 |
| Yes | 30.8 | 24.6 |  |
| No | 69.2 | 75.4 |  |
| Body mass index^4^ |  |  | 0.01 |
| Normal weight (<25 kg/m^2^) | 75.8 | 56.3 |  |
| Overweight (25-29.9 kg/m^2^) | 11.3 | 23.0 |  |
| Obesity (≥30 kg/m^2^) | 12.9 | 20.7 |  |
| Any alcohol consumption |  |  | 0.08 |
| Yes | 74.1 | 79.3 |  |
| No | 26.0 | 20.7 |  |
| ≥ 30 minutes of moderate- or vigorous-intensity physical activity per day |  |  | 0.01 |
| Yes | 26.0 | 34.6 |  |
| No | 74.1 | 65.4 |  |

^1^ number of participants differs due to missing data

^2^ p-values from chi-square tests

^3^ Other cultural or ethnic groups include African, Middle Eastern, South American, Central American and Caribbean Islander

^4^ Women with underweight (BMI <18.5 kg/m^2^) were included in the normal weight category due to low numbers
