# Supplementary material for: Association between pregnancy intention and psychological distress among women exposed to different levels of restrictions during the COVID-19 pandemic in Australia
Source: PLoS One. 2022 Aug 25;17(8):e0273339. doi: 10.1371/journal.pone.0273339 (PMC9409515; doi:10.1371/journal.pone.0273339)
Supplement: S3 Table — (DOCX) [file pone.0273339.s004.docx]

**S3 Table.** Associations between pregnancy intention and psychological distress by level of COVID-19 transmission rates and lockdown restrictions based on complete case analysis (non-imputed data), N = 560

|  | Outcome: log-transformed psychological distress score | |  | | Outcome: high/very high vs low/moderate psychological distress | | |  |
| --- | --- | --- | --- | --- | --- | --- | --- | --- |
|  | Unadjusted  Coefficient (95% CI) | Adjusted^1^  Coefficient (95% CI) | |  | | Unadjusted  Odds ratio (95% CI) | Adjusted^1^  Odds ratio (95% CI) | |
| *Overall study population, n = 560* | | | | | | | |  |
| Not planning to become pregnant | Reference | Reference | |  | | Reference | Reference | |
| Planning to become pregnant | **0.09 (0.01, 0.17)** | 0.04 (-0.03, 0.12) | |  | | **1.65 (1.09, 2.50)** | 1.33 (0.83, 2.13) | |
|  |  |  | |  | |  |  | |
| *Women exposed to high viral transmission rates and strict lockdown restrictions, n = 125* | | | | | | | |  |
| Not planning to become pregnant | Reference | Reference | |  | | Reference | Reference | |
| Planning to become pregnant | **0.20 (0.04, 0.35)** | **0.19 (0.02, 0.36)** | |  | | **2.67 (1.16, 6.15)** | 3.08 (0.98, 9.70) | |
|  |  |  | |  | |  |  | |
| *Women exposed to low viral transmission rates and less strict lockdown restrictions, n = 435* | | | | | | | |  |
| Not planning to become pregnant | Reference | Reference | |  | | Reference | Reference | |
| Planning to become pregnant | 0.05 (-0.04, 0.15) | 0.01 (-0.08, 0.10) | |  | | 1.39 (0.86, 2.24) | 1.12 (0.65, 1.93) | |

^1^ Adjusted for age group, marital status, location (overall study population only), highest level of education completed, annual household income before tax, food affordability, employment prior to the pandemic, number of children in the household, alcohol consumption, body mass index
