# Supplementary material for: Association between pregnancy intention and psychological distress among women exposed to different levels of restrictions during the COVID-19 pandemic in Australia
Source: PLoS One. 2022 Aug 25;17(8):e0273339. doi: 10.1371/journal.pone.0273339 (PMC9409515; doi:10.1371/journal.pone.0273339)
Supplement: S1 File — (PDF) [file pone.0273339.s005.pdf]

A1. Background Information Screenout

Food, exercise and mind survey during COVID 19 pandemic in women

By Monash Centre for Health Research and Implementation at Monash University

Invitation to participate

We are interested in learning about the impact of the COVID 19 pandemic has on food intake, physical activity and mental health of women and their infants. This study is voluntary. If you decide to participate but change your mind, you can discontinue at any time. However, data collected up to the time you withdraw will still form part of the research project results.

What is this project about and why is it being conducted?

This national survey aims to understand the impact of COVID 19 pandemic on food intake, physical activity and mental health of women in Australia. Some Australians are already experiencing negative impact but we do not have a complete picture of who is affected and how much. We need to know more so that targeted help could be offered to those who need it.

What are we asking of you?

If you are 18 or older, we would like you to participate in a 15-minute survey. The survey will ask you around food intake, physical activity, body weight and mental

wellbeing. If you just had a baby, you will also be asked about infant feeding.

What are the benefits of doing the survey?

There are no direct benefits to you from completing the survey. However, your participation in the survey will help us understand how the COVID 19 pandemic is affecting the diet, physical activity and mental wellbeing of women in Australia.

Your confidentiality is our priority

All information collected as part of the survey will be kept safe and secure on a password-protected computer. After seven years from the final publication of the research project, the stored files will be destroyed.

What are the risks to you with participating?

It is unlikely that participating in this study will cause any stress over and above other stressors in your life. However, you may feel some discomfort from answering questions related to mental health. You can skip questions or exit the survey at any time. Your consent to participate will be implied if you choose to complete this survey.

If any mental health concerns arise due to the survey, please contact one of the following free mental health services below:

Lifeline Australia - 13 11 14 (https://www.lifeline.org.au/)
Blue Knot Foundation - 1300 657 380 (https://www.blueknot.org.au/)
Beyond Blue - 1300 22 4636 (https://www.beyondblue.org.au/)
Mental Health Online - https://www.mentalhealthonline.org.au/

Consent to Participate

By clicking the “Next” arrow at the bottom of the page, you are indicating that you are at least 18 years old and that you consent to participate in this research.

Further information about the study

For further information about the study or if you have questions, please contact Chief Investigator Siew Lim at siew.lim1@monash.edu

The current study has been approved by the Monash University Human Research Ethics Committee (MUHREC project: xxxxx). Should you have any concerns or complaints about the conduct of the project, you are welcome to contact the Executive Officer, Monash University Human Research Ethics Committee (MUHREC): Tel: +61 3 9905 2052; Fax: +61 3 9905 3831; Email: muhrec@monash.edu; Address: 26 Sports Walk, Clayton Campus Research Office, Monash University VIC 3800

If you experience display issues with the survey, please try a different device and/or web browser.

What is your postcode?

What state or territory are you from?

- Victoria
- Queensland
- New South Wales
- Tasmania
- Northern Territory

- ○ Western Australia
- ○ Australian Capital Territory
- ○ South Australia

What is your sex?

- ○ Male
- ○ Female
- ○ Other
- ○ I prefer not to say

What is your age group?

- ○ Under 18
- ○ 18-24
- ○ 25-34
- ○ 35-44
- ○ 45-50
- ○ Over 50

A2. Background information Continued

How would you describe your cultural and ethnic group (optional)?

- ○ Oceanian (Australian peoples, New Zealand peoples, Pacific Islanders)

- North-west European (British, Irish, Western European, Northern European)
- Southern and Eastern European (Southern European, South Eastern European, Eastern European)
- North African and Middle Eastern (Arab, Jewish, Peoples of the Sudan, other North African and Middle Eastern)
- South East Asian
- North East Asian
- Southern and Central Asian
- North American
- South American
- Central American
- Caribbean Islander
- Central and West African
- Southern and East African
- I prefer not to say

What is your highest level of completed education?

- Primary/elementary school or less
- Secondary/high school
- TAFE
- University Degree
- Graduate/Postgraduate degree
- I have never been to school
- I don't know/I prefer not to answer

Before the Covid 19 pandemic, how would you describe your employment status?

- Retired
- Homemaker
- Government assistance
- Government disability support
- Student
- Part-time employment
- Full-time employment
- Casual employment
- I don’t know/I prefer not to answer

Has your employment status changed since the Covid 19 pandemic?

- Yes
- No

If yes, how would you describe your current employment status?

- Retired
- Homemaker
- Government assistance e.g. family payments
- Government assistance e.g. Job Keeper
- Government assistance e.g. Job Seeker
- Government disability support
- Student
- Part-time employment
- Full-time employment
- Casual employment

- I don’t know/I prefer not to answer

What is your annual household income before tax?

- $0-$24,999
- $25,000-$49,999
- $50,000-$74,999
- $75,000-$99,999
- $100,000-$124,999
- $125,000-$149,999
- >$150,000
- I don't know/I prefer not to answer

What is your current living circumstance?

- Own home
- Rented home
- Living with family
- Emergency accommodation (hostel, B&B, hotel)
- I prefer not to say

How many adults and children live with you at home currently?

Adults [ ]

Children

If you do have children living with you at home, what are the ages of your children?
Please enter their ages in whole numbers, separated by commas (e.g. 7, 10, 4)

Ages of your children

What is your current marital status?

- Single
- Married or de facto
- I prefer not to say

What is your intention around pregnancy?

- I am not planning to become pregnant
- I am planning to become pregnant
- I am currently pregnant
- I just had a baby in the last 12 months
- I don't know/I prefer not to answer

(If yes) How many **months** ago was the baby born?

Have you ever had, been diagnosed or treated for (tick all that apply):

- ☐ Polycystic ovary syndrome
- ☐ Infertility
- ☐ Menopause
- ☐ Type 2 diabetes
- ☐ Diabetes onset in pregnancy
- ☐ Hypertension onset in pregnancy
- ☐ Pre-eclampsia
- ☐ Preterm birth delivery
- ☐ Delivery of a baby before 38 weeks of pregnancy
- ☐ Delivery of a baby with body weight of <2500g after 38 weeks of pregnancy
- ☐ None of the above
- ☐ I don't know/I prefer not to answer

B. U.S. HOUSEHOLD FOOD SECURITY SURVEY MODULE

These next questions are about the food eaten in your household in the last 6 months and whether you were able to afford the food you need.

Which of these statements best describes the food eaten in your household in the last 6 months:

- Enough of the kinds of food we want to eat
- Enough but not always the kinds of food we want
- Sometimes not enough to eat
- Often not enough to eat
- I don't know/I prefer not to answer

For the next several statements about food situation, please answer whether the statement was often true, sometimes true, or never true for your household in the last 6 months:

“(I/We) worried whether (my/our) food would run out before (I/we) got money to buy more.”
Was that often true, sometimes true, or never true for (you/your household) in the last 6 months?

- Often true
- Sometimes true
- Never true

- I don't know/I prefer not to answer

"The food that (I/we) bought just didn't last, and (I/we) didn't have money to get more."
Was that often, sometimes, or never true for (you/your household) in the last 6 months?

- Often true
- Sometimes true
- Never true
- I don't know/I prefer not to answer

"(I/we) couldn't afford to eat balanced meals."
Was that often, sometimes, or never true for (you/your household) in the last 6 months?

- Often true
- Sometimes true
- Never true
- I don't know/I prefer not to answer

B1. Adult Stage 2: Questions AD1-AD4

In the last 6 months, since last (name of current month), did you or other adults in your household ever cut the size of your meals or skip meals because there wasn't enough money for food?

- Yes
- No
- I don't know/I prefer not to answer

[IF YES ABOVE] How often did this happen—almost every month, some months but not every month, or in only 1 or 2 months?

- Almost every month
- Some months but not every month
- Only 1 or 2 months
- I don't know/I prefer not to answer

In the last 6 months, did you ever eat less than you felt you should because there wasn't
enough money for food?

- Yes
- No
- I don't know/I prefer not to answer

In the last 6 months, were you ever hungry but didn't eat because there wasn't enough
money for food?

- Yes
- No
- I don't know/I prefer not to answer

In the last 6 months, did you lose weight because there wasn't enough money for food?

- Yes
- No
- I don't know/I prefer not to answer

B2. Adult Stage 3: Questions AD5 and AD5a

In the last 6 months, did (you/you or other adults in your household) ever not eat for a
whole day because there wasn't enough money for food?

- Yes
- No
- I don't know/I prefer not to answer

[IF YES ABOVE, ASK] How often did this happen—almost every month, some months
but not every month, or in only 1 or 2 months?

- Almost every month
- Some months but not every month
- Only 1 or 2 months
- I don't know/I prefer not to answer

B3. Child Stage 1: Questions CH1-CH3

The next several statements have been made by people about the food situation of their children.

For these statements, please answer whether the statement was OFTEN true, SOMETIMES true, or NEVER true in the last 6 months for your children living in the household who are under 18 years old.

“I relied on only a few kinds of low-cost food to feed my child/the children

because we were running out of money to buy food.”
Was that often, sometimes,
or never true for your household in the last 6 months?

- Often true
- Sometimes true
- Never true
- I don't know/I prefer not to answer

“I couldn’t feed my child/the children a balanced meal, because we
couldn’t afford that.” Was that often, sometimes, or never true for your household
in the last 6 months?

- Often true
- Sometimes true
- Never true
- I don't know/I prefer not to answer

"My child was/The children were not eating enough because we just couldn't
afford enough food." Was that often, sometimes, or never true for your household
in the last 6 months?

- Often true

- Sometimes true
- Never true
- I don't know/I prefer not to answer

B4. Child Stage 2: Questions CH4-CH7

In the last 6 months, did you ever cut the size of your child's/any of the children's meals because there wasn't enough money for food?

- Yes
- No
- I don't know/I prefer not to answer

In the last 6 months, did any of the children ever skip meals because
there wasn't enough money for food?

- Yes
- No
- I don't know/I prefer not to answer

[IF YES ABOVE ASK] How often did this happen—almost every month, some months

but not every month, or in only 1 or 2 months?

- ○ Almost every month
- ○ Some months but not every month
- ○ Only 1 or 2 months
- ○ [ ] DK

In the last 6 months, was your child/were the children ever hungry but you just
couldn't afford more food?

- ○ Yes
- ○ No
- ○ I don't know/I prefer not to answer

In the last 6 months, did your child/any of the children ever not eat for a whole day
because there wasn't enough money for food?

- ○ Yes
- ○ No
- ○ I don't know/I prefer not to answer

C. Food intake

How often do you usually have breakfast?

- ◯ Never
- ◯ 1-2 a week
- ◯ 3-4 a week
- ◯ 5-6 a week
- ◯ Everyday
- ◯ I don’t know/I prefer not to answer

How often do you eat fruit, excluding fruit juice?

- ◯ Once or more a day
- ◯ 4 to 6 times a week
- ◯ 1 to 3 times a week
- ◯ Less than once a week
- ◯ Never
- ◯ I don’t know/I prefer not to answer

(If option 1-4) How many portions a day on average do you eat, on the days you do eat fruit? A portion is a medium apple, a pear, orange or similar-sized fruit, two small fruits like mandarin, or a cup of fruit like grapes

- ◯ [ ] Number of portions daily (1-99)
- ◯ I don’t know/I prefer not to answer

How often do you eat vegetables or salad, excluding juice and potatoes?

- ○ Once or more a day
- ○ 4 to 6 times a week
- ○ 1 to 3 times a week
- ○ Less than once a week
- ○ Never
- ○ I don't know/I prefer not to answer

(If option 1-4) How many portions a day on average do you eat, on the days you do eat vegetables? A portion is ½ cup cooked or 1 cup raw vegetables.

- ○ [ ] Number of portions daily (1-99)
- ○ I don't know/I prefer not to answer

How often do you eat lean meat and poultry, fish, eggs, nuts/seeds, or legumes/beans?

- ○ Once or more a day
- ○ 4 to 6 times a week
- ○ 1 to 3 times a week
- ○ Less than once a week
- ○ Never
- ○ I don't know/I prefer not to answer

(If option 1-4) How many portions a day on average do you eat, on the days you do eat vegetables? A portion is ½ cup cooked or 1 cup raw vegetables.

- ○ [ ] Number of portions daily (1-99)
- ○ I don't know/I prefer not to answer

How often do you have dairy or alternative products?

- ○ Once or more a day
- ○ 4 to 6 times a week
- ○ 1 to 3 times a week
- ○ Less than once a week
- ○ Never
- ○ I don't know/I prefer not to answer

(If option 1-4) How many portions a day on average do you eat, on the day you do have dairy or alternative products? A portion is 1 cup milk (including calcium-added milk alternatives), ¾ cup yoghurt or 2 slices of cheese.

- ○ [ ] Number of portions daily (1-99)
- ○ I don't know/I prefer not to answer

How often do you have 'treat' foods (including ice-cream, processed meat eg sausage and ham, cracker, sweet biscuit, pastry, doughnut, cake or muffin, jam, honey, lollies, chocolate, cream, butter/margarine, pie, chips)?

- ○ 1-2 a day
- ○ 3-5 a day
- ○ 5-7 a day
- ○ 7-10 a day

- >10 a day
- Don't eat every day
- Never
- I don't know/I prefer not to answer

(If option 1-5) How many portions a day on average do you eat, on the day you do eat 'treat' foods?

- [ ] 2 scoops of ice cream (1-99)
- [ ] 2 slices of processed meats eg salami, mettwurst etc (1-99)
- [ ] 2 thin sausages (1-99)
- [ ] 30g crackers (1-99)
- [ ] 2-3 sweet biscuits (1-99)
- [ ] 1 regular-sized doughnut (1-99)
- [ ] 1 slice of cake or 1 cupcake or sweet muffin (1-99)
- [ ] 1 tablespoon jam/honey (1-99)
- [ ] 5-6 lollies (1-99)
- [ ] 1 small bar (25g) of chocolate (1-99)
- [ ] 2 tablespoon cream (1-99)

- [ ] [ ] 1 tablespoon butter or margarine (1-99)
- [ ] [ ] 1/3 (60g) individual serve pie (1-99)
- [ ] [ ] 12 (60g) fried hot chips (1-99)
- [ ] I don't know/I prefer not to answer

How often do you have sugar-sweetened beverages (including soft drink, flavoured milk, energy drink, fruit drink, hot beverages with added sugar)?

- ○ Once or more a day
- ○ 4 to 6 times a week
- ○ 1 to 3 times a week
- ○ Less than once a week
- ○ Never
- ○ I don't know/I prefer not to answer

(If option 1-4) How many portions a day on average do you have, on the day you do have sugar-sweetened beverages? A portion is 375ml (1 can) of beverage.

- ○ [ ] Number of portions daily (1-99)
- ○ I don't know/I prefer not to answer

How often do you have alcohol?

- ○ 4 times or more a week
- ○ 2 to 3 times a week

- 2-4 times a month
- Monthly or less
- Never
- I don’t know/I prefer not to answer

(If option 1-4 in Q8) How many portions a day on average do you have, on the day you do have alcohol? A portion is 200ml wine, 60 ml spirits, 600 ml light beer, 400 ml regular beer

- 1-2 drinks
- 3-4 drinks
- 5-6 drinks
- 7-8 drinks
- 9+ drinks
- I don’t know/I prefer not to answer

How much would you typically spend on grocery shopping (food only) in a week?

- <100 dollars
- 100-200 dollars
- 200-400 dollars
- >400 dollars
- I don’t know/I prefer not to answer

Is getting fresh fruit and vegetables a problem in your area?

- A big problem
- A bit of a problem
- Not a problem
- I don't know/I prefer not to answer

D. Physical activity

Please state HOW MANY TIMES you did each type of activity LAST WEEK

Only count activities that lasted for 10 minutes or more

*(If you did **NOT** do an activity, please type '0')*

	Number of Times
Walking briskly (for recreation or exercise, or to get from place to place)	
Moderate leisure activity (like social tennis, moderate exercise classes, recreational swimming, dancing)	
Vigorous leisure activity (that makes you breathe harder or puff and pant like aerobics, competitive sport, vigorous cycling, running, swimming)	
Vigorous household or garden chores (that make you breathe harder or puff and pant)	

Please state the TOTAL TIME you spent altogether doing each type of activity LAST WEEK

Add up all the times you spent in each activity to get the total time for each activity

	Hours	Minutes
Walking briskly (for recreation or exercise, or to get from place to place)		
Moderate leisure activity (like social tennis, moderate exercise classes, recreational swimming, dancing)		
Vigorous leisure activity (that makes you breathe harder or puff and pant like aerobics, competitive sport, vigorous cycling, running, swimming)		
Vigorous household or garden chores (that make you breathe harder or puff and pant)		

Please estimate how much time you spent SITTING in each of the following activities on your last

WORKING day

	Hours	Minutes
For transport (e.g. in car, bus, train etc)		
At work (eg sitting at a desk or using a computer)		
Watching TV		
Using a computer at home (email, games, information, chatting)		
Other leisure activities (socialising, movies etc, but NOT including TV or computer use)		

Please estimate how much time you spent SITTING in each of the following activities on your last NON-WORKING day (weekend day or day off)

	Hours	Minutes

	Hours	Minutes
For transport (e.g. in car, bus, train etc)		
At work (eg sitting at a desk or using a computer)		
Watching TV		
Using a computer at home (email, games, information, chatting)		
Other leisure activities (socialising, movies etc, but NOT including TV or computer use)		

E. Weight

What is your current weight? _ _ _ _kg

- _kg
- I don't know/I prefer not to answer

What is your current height? _ _ _ _m

- _m
- I don't know/I prefer not to answer

Since the Covid 19 pandemic, have you gained or lost weight?

- Gained
- Lost
- Stayed the same
- I don’t know/I prefer not to answer

(If option i or ii) How much did it change? _ _ _kg

- [ ] _kg
- I don't know/I prefer not to answer

F. Kessler Psychological Distress Scale (K10)

Please tick the answer that is correct for you:

	All of the time (score 5)	Most of the time (score 4)	Some of the time (score 3)	A little of the time (score 2)	None of the time (score 1)	I prefer not to answer
1. In the past 4 weeks, about how often did you feel tired out for no good reason?	○	○	○	○	○	○
2. In the past 4 weeks, about how often did you feel nervous?	○	○	○	○	○	○

	All of the time (score 5)	Most of the time (score 4)	Some of the time (score 3)	A little of the time (score 2)	None of the time (score 1)	I prefer not to answer
3. In the past 4 weeks, about how often did you feel so nervous that nothing could calm you down?	○	○	○	○	○	○
4. In the past 4 weeks, about how often did you feel hopeless?	○	○	○	○	○	○
5. In the past 4 weeks, about how often did you feel restless or fidgety?	○	○	○	○	○	○
6. In the past 4 weeks, about how often did you feel so restless you could not sit still?	○	○	○	○	○	○
7. In the past 4 weeks, about how often did you feel depressed?	○	○	○	○	○	○
8. In the past 4 weeks, about how often did you feel that everything was an effort?	○	○	○	○	○	○
9. In the past 4 weeks, about how often did you feel so sad that nothing could cheer you up?	○	○	○	○	○	○

	All of the time (score 5)	Most of the time (score 4)	Some of the time (score 3)	A little of the time (score 2)	None of the time (score 1)	I prefer not to answer
10. In the past 4 weeks, about how often did you feel worthless?	○	○	○	○	○	○

Powered by Qualtrics
